# Rhoticity in English, a Journey Over Time Through Social Class: A Narrative Review

**DOI:** 10.3389/fsoc.2022.902213

**Published:** 2022-05-06

**Authors:** Davide Costa, Raffaele Serra

**Affiliations:** ^1^Department of Law, Economics and Sociology, University Magna Graecia of Catanzaro, Catanzaro, Italy; ^2^Department of Medical and Surgical Sciences, University Magna Graecia of Catanzaro, Catanzaro, Italy; ^3^Department of Social Sciences, Vitambiente, Catanzaro, Italy

**Keywords:** sociology, sociolinguistics, social class, rhoticity, dynamics of class

## Abstract

Rhoticity in English refers to the pronunciation of the consonant /r/ in all r position contexts, while non-rhoticity refers to the dropping of the /r/ sound in particular r positions. In this context, the two English varieties, classified as rhotic and non-rhotic can be found both in British and American English-speaking people, but also in other English-speaking countries. The most updated information about rhoticity, related history of classes in the English-speaking people have been retrieved from the most important database such as ScienceDirect and Scopus. Society and language are strictly related, especially in rhoticity changes that occurred over time in the English-speaking people. In fact, rhoticity is a dynamic sociolinguistic phenomenon as it was influenced by social class changes during centuries, and even now it is constantly evolving. Rhoticity is also connected to social mobility in English-speaking countries and is also an indicator of social displacement from one social class to another. In fact, class, language, and social differentiation are only the terms of an inseparable social equation. In conclusion, in the dynamics of class, rhoticity and non-rhoticity seem are related to socio-anthropological issues that confirm an intimate connection with the process of social differentiation.

## Introduction

Rhotic varieties in English are the pronunciation of the consonant /r/ in all r position contexts (word-initially word-medially, and word-finally), while other varieties of English language are classified as non-rhotic. In non-rhotic varieties, speakers do not pronounce /r/ when it is at the end of a word or in postvocalic environments, that is, when it is immediately after a vowel and not followed by another vowel. Rhotic and non-rhotic pronunciations can be found both in Received Pronunciation (RP) (standard British English pronunciation) and in General American (GA) pronunciation (standard American English pronunciation). Non-rhotic countries are England, especially the south-west; Wales; New Zealand, Australia, South Africa, Black Africa, the Caribbean, except for Barbados; the American southern states, the Boston area of New England, and New York City vernacular speech; and Black English Vernacular in the US. The main rhotic countries are US (the northern and western states of the US apart from the Boston area and New York City), Canada, India, Ireland, south-western England, Scotland, and Barbados. All English accents were rhotic up until the early Modern English period and non-rhoticity variety was a relatively late development. It is said to have started in 18th century as a prestige motive in socio cultural contexts in British culture (Demizeren, 2012, p. 2660; Boyce et al., 2016, p. 3; Villarreal et al., 2020, p. 24). The advent of radio and television in the 20th century established a national standard of American pronunciation that preserves historical /r/, with rhotic speech in particular becoming prestigious in the United States rapidly after the Second World War (Labov et al., 2006, p. 5, 8, 11–14). The aim of this article is to study the social factors that influenced rhoticity in English.

## Main Objectives

The main purpose of this review article is to analyze, with the methodological approaches typical of sociology, the phenomenon of rhoticity; it's a particular phenomenon that which it falls in the scope of the liquid consonants (Proctor et al., 2019, p. 1). Therefore, the second purpose is aimed at a qualitative analysis, to try to broaden, through new theoretical presuppositions, the study of such a peculiar phenomenon. The last, but no less important purpose, consists in the attempt to implement the attention both on the use of language in sociological terms, and on the variations that occur in it.

## Methodology

The most updated information about rhoticity, related history of classes in the English-speaking people have been retrieved from the most important database such as ScienceDirect and Scopus.

Search terms used were rhoticity in English; rhoticity and social class; rhoticity and sociolinguistics; rhoticity and sociology; language and dynamic of class. Inclusion criteria were all articles dealing with search terms used, no time frame selected. We also reviewed the reference lists of retrieved studies to identify studies that had not been identified by the search strategy.

## Results

### Sociology and Sociolinguistics

Language has always been one of the main discriminating variables between man and animal. Language, in these terms, is the way in which to enter a relationship with the other. This is therefore the reason why sociology, as a science that deals with what is human, has always been concerned with the study of language; it is no coincidence that there is a sociological branch that is interested in it, namely the sociology of language.

Very often, in various fields there is a tendency to associate, in terms of synonyms, this field of research with sociolinguistics, but “the examination of a sample of texts that go under this title should be sufficient to show that sociolinguistics touches only a fraction of the fields of investigation a) and b) and brings little or no interest to field c). Sociolinguistics—therefore—deals above all with variations in the structure of the use of language in relation to variations in the social context, while sociology of language is more concerned with the analysis of the social functions of language, expressive and regulating, in all their manifestations” (Gallino, 2014, p. 407).

In summary, we could argue that sociology studies society through language, while sociolinguistics studies language in society.

Since sociology, and not only sociology, marries the pluralism of ideas, it is preferable to go beyond these definitional limitations, which, as often happens, only tend to make any type of argument fallacious and complex.

Surely society and language are strictly interdependent, one reflecting the other, or rather, language is a clear example of the “tangible” transposition of society.

In this regard, Ludwig Wittgenstein in the “Tractacus logico-philosophicus”, recalls how a set of activities and situations in which each individual is inserted as part of a specific society, can be understood as a form of life, for which language is a part of a form of life and therefore language is the means that the people use to understand each other in relation to the activities in which they are involved, and it expresses the form of social life. In this way, it is the means through they interpret.

It should also be remembered that sociology and linguistics have established themselves in mutual indifference, while the sociology of language is it has long been a much-neglected branch of sociology; moreover, the age-old problem for which linguistics, in many cases, has neglected the analysis of sociological aspects. This mutual indifference would be attributable to some situations:

a) the sociological recognition of the task of language in social agglomerations; and precisely because social scientists they considered language as an unfailing principle necessary for each group, they felt that language was attributable to behavioral differentiation and have significantly neglected the theoretical-empirical analysis.

b) also, for the social role of the linguistic code, or rather on the relationship that it establishes with society, so that language would seem to define boundaries that are arbitrary and conventional, i.e., implicitly social.

This initial differentiation between the two disciplines, typical of the beginning of the last century, underwent a change, starting from the sixties, when many linguists began to exalt the social sphere of language and, on the other hand, many social scientists began to detect the relapses, of the social nature, of the language. This initial convergence found its own explanation or the reciprocal orientation toward attitude in all its social facets.

All this has led to a new awareness, especially in sociological knowledge, or rather in modern societies there are a plurality of attitudes linguistics in various geographical contexts, or a specific class. In particular sociologists begin to grasp as the causes linguistics of the scholastic failure of children of lower classes (Bernstein, on the process of formation of groups social (Barth, Blom and Gumperz), on the relationship between systems cultural and social systems (Bernstein), on face-to interaction face (Goffman), linked to different theoretical approaches such as interactionism symbolic, ethnomethodology, the critical school of Frankfurt (Habermas), which underline the crucial role of symbolism in social action.

### Some Sociological Aspects of Language

The sociological question of language mainly concerns the process of interpretation, which leads us to quote an important exponent of sociology, namely George Mead, father of the so-called symbolic interactionism, in which language constitutes an essential element, since it incorporates signs and symbols to which specific meanings are attributed. These meanings are shared by several subjects, for which language is the social “fact” for excellence since the social actor is insofar as he has a self, whose form is made possible by his ability to use a common language. This means that our mind, again according to Mead, has the ability to process thought, only thanks to the process of social interaction mediated symbolically, that is, through language.

If language is all this, it is at the same time the product and producer of social change, in the sense that the power of time and its constant flow sediments in language; in this regard, the philosopher Heidegger defining man (Being-there in his “vocabulary”) understood in his possibility and ability of being- he is time itself and is not in time, remains as evidence that time has an intimate relation with language. Symbolic mediation is absolutely central (Heidegger, 1947).

Another important exponent of sociology has understood the relevance of language, namely Habermas who supports the importance of symbolic mediation since only thanks to it, men can be linked with each other in search of the mutual ability to understand each other; it is in this direction that the sociologist moves to the point of defining language as the founding requirement of the perpetration of social life.

The position of Saussure (1974) finds an important connection to rhoticity, since he favors orality considering it central in social agglomerations, just as it is, in more advanced societies, the variation to which languages are subjected from the point of view of space-thunderstorm; languages such as English, in particular, are subjected to a double and fixed pressure both for the influence of the cultures with which a population comes into contact (think, as we shall see, of the case of rhoticity in America), and for the constant need to change the vocabulary.

From this point of view, the phonetic transformations of English offer a very significant exemplification: not only for the continuous influx of neologisms that make us seem atavistic already the language of half a century ago, but it is above all the phenomenon of rhoticity to be particularly interesting; phenomenon that falls within the linguistic phonetic repertoire that reverberates in social action.

Thus, the sociology of language reminds us that linguistic changes are not located only in time and space (Gadamer, 1960). Many variations depend on the social sphere, or on the positioning of speakers in the social space: even if unconsciously, our speeches continuously offer signals, which are revealing of our social and geographical identity (think of the geographical distribution of rhoticity), but also the image we have of ourselves and, moreover, how we want to appear to others. The diversity and variety of the social plot and social heterogeneity has greatly increased thanks to the transition from agricultural to industrial societies (as we will see in the next paragraph on the history of the phenomenon in question); this is reflected in the linguistic variability: almost all societies have heterogeneous languages, composed of many variants, each of which plays different roles and is subjected to different collective actions, judgments and attitudes; in this regard, we will see how, according to our hypotheses, this aspect is precisely the event underlying the rhotic/non rhotic dichotomy.

All this leads us to remember how linguistic variation reflects the social position of the speaker, but it can serve, and it is, usually, used, also to establish, or change pre-existing relationships: which is of particular interest to us, as is well known.

This appears clear in the distinction between rhotic and non-rhotic from which we find a certain correspondence between the class of origin and the phonetic approach used: in the tendency to social ascent, particularly in the middle classes, it is common to find hyper-correctisms or the use of forms linguistics typical of higher social strata in an attempt to appropriate, symbolically at least, higher status as Veblen argues: those who belong to the lower middle class (petty bourgeoisie, clerical classes, small provincial professionals) speak a formally very rigorous and correct language, sometime refined expressions (with different kind of grotesque effects). It is clear that, in these cases, the use of language, is the one hand the desire to differentiate from the poor class, on the other the desire to be accepted by the upper classes to which one aspires to belong.

It is still necessary to insist on some important theoretical passages for the purposes of this article, in this regard, Saussure (1974, p. 28–30) distinguishes an area in the individual context of communicating (the so-called word act) from a more social and systematic or langue, deeply inserted into the society of which it express's needs, interests, values; the phenomenon related to rhoticity, therefore, it would concern langue; in this regard, in the work by Saussure, the cognition of the sociality of language is constant and the way in which a linguistic system is structured according to the social agglomeration of speakers. In this way society as the foundation of the system and of meaning must be placed in a privileged position because the conception in which the value of the sign, that is its formal and semantic identity, is entirely entrusted to the system, a second conception is emerging that it makes a second variable intervene, society: It is only the social fact that creates what exists in a semiological system.

But it is with Berger and Luckmann that it is possible to understand how language is essential since “A whole world can be actualized at any time by means of language… a whole world it can be opened in front of me at any moment… language 'makes it present for me not only individuals who are physically absent at that time, but also people who belong to the past remembered or reconstructed, and people projected as imaginary figures in the future” (Beger and Luckman, 1989, p. 62–3). For the two sociologists, linguistic mediation is the essential prerequisite for being able to communicate, but also and perhaps above all to be able to be in the social world. Here, then, is the sociological root, at least in part, of the phenomenon of rhoticity, because by choosing to pronounce a consonant or not, a position is affirmed, a certain degree of prestige, and ultimately, a socio-spatial position.

This is closely linked to the philosophy of language according to which language plays an essential role in the structuring of thought and therefore the relationship with reality. That is: Logos and Epos, being and essence, entity and existing.

The story of rhoticity/non-rhoticity is a typical example of class struggle. As Demizeren (2012, p. 2660) or Jauriberry (2020, p. 2) recalls, from a historical point of view languages such as Celtic, Roman, Germanic were rhotic, in fact they left a phonetic-auditory imprint in the various English dialects.

In this regard, we must remember that the Celts were among the first peoples to settle in Europe, so their rhotic language was predominant for a long time.

According to various sources, from the first century BC. until the fifth century AD, the Romans colonized England and in general a large part of Europe until 476 AD (Demizeren, 2012, p. 2660); the language of the Roman Empire, however, was Latin, i.e. a Rhotian language whose r was an alveolar trill /r/ (Meer et al., 2021, p. 2).

English also officially established itself when the Germanic tribes and their linguistic-cultural heritage reached the British Isles in 449 (Gelderen, 2006, p. 1). In the 9th century, the Scandinavian Vikings invaded England, who spoke Old Norse, which derives from the same ancestral Proto-Germanic language from which Anglo-Saxon had originated.

In 1,066, then, the Scandinavian and Norman conquest brought a clear trill /r/, which was maintained until the 18th century in England (Jauriberry, 2020, p. 3). In particular, this event brought a huge number of French words in Norman form that contained a highly audible trill /r/, which survived until the first phase of modern English to be stated during the mid-1400 s as claimed by Wyld (1920) and Jespersen (1954). For Wyld, the loss of r began in eastern England in the mid-15th century, and by the mid-16th century it had spread to both other consonants and the London vernacular. Hill (1940), on the other hand, describes a loss of the pronunciation of /r/ in the 14th century.

With the various attempts of colonization of America by England, a significant implantation of the non-Roman accent was established in these places too: in North America, in particular, in eastern New England and the coastal areas from Virginia up to the extreme south of Carolina, they were, in fact, initially colonized by the south-east area of England, where the weakness was more affirmed. In addition, western New England and the mountainous interior were later colonized by both Scots-Irish, who were totally rhotic, and by people from the northern and western parts of Britain who may have retained more pronounced /r/.

During the mid-1700s, postvocalic /r/ was still employed, but by the mid-1700s it was not used, especially after open/low vowels (Jauriberry, 2020, p. 4).

During the early nineteenth century, the southern British was transformed into the non-rhotic variety, although some varieties were persistent until the second half of the nineteenth century.

The loss of postvocal /r/ in British English began to influence the accents used in the seaside cities of South and East America, which had significant socio-economic interactions with England; in this way the pronunciation of the upper middle class became non-rhotical while the other social classes remained rhotic. With respect to this last aspect, Kurath-McDavid recalls the fact that a weak pronunciation of /r/ is a prestigious feature in southern England, and therefore, any /r/ of the variant present in the first English coastal colonies would be attributable to the people of American urban society and plantations.

The loss of rhoticity has been more correctly dated by historians to the 18th century.

In this regard, as Bailey (1996, p. 100), Trudgill (2006, p. 10) and recently Jauriberry (2020, p. 4) recall, the passage from consonant r to vowel r, even if it was sporadic previously, instead acquired strength toward the end of the eighteenth century; Strang (1970, p. 112), confirming this, recalls the fact that this progressive weakening established itself starting in the seventeenth century and was then reduced to a vowel segment at the beginning of the eighteenth century.

This is the reason why Canadian English, Irish English and American English are predominantly rhotic, because the English language was exported to these colonial areas from Britain earlier, but also during the 17th century, that is, before the process of loss of rhoticity began in Great Britain, and that the British in the southern hemisphere were non rhotic, as English was exported to these areas in the 19th century, that is, after the loss of rhoticity (Trudgill, 1984). This aspect is so tangible that no one has ever thought to question it, as Trudgill (2006) recalls.

In this regard, very useful is Ellis's (1889, p. 485) research on dialects in England in the twentieth century brought to light the fact that the areas where rhoticity was not recorded, in the 1860s.

Starting from the twentieth century, however, there has been a progressive reduction in rhoticity throughout England.

To confirm this, the Dialect Survey was carried out in the 1950s (Orton and Barry, Orton and Barry, 1969–1971), which ascertained not only a real attenuation of rhoticity, but also a significant shift to the west.

Chambers and Trudgill (1998, p. 95) further demonstrated that the degree of reduction in pronunciation was even more reduced than Orton and Barry were able to document (Asprey, 2007, p. 82).

On the phenomenon of the reduction of rhoticity, Sullivan (1992), in his survey documented only 8% of rhotic subjects out of a sample of individuals belonging to the middle class.

Britain (2002, p. 52) recalled, in this regard, the research of Dudman (2000), with which this trend was demonstrated since the degree of gearing in adolescents, belonging to the working class, born in 1987 was about half compared to subjects born between 1906 and 1924.

The reduction today more than ever is more and more accentuated, in fact Asprey (2007, p. 99) argues that the reduction trend continues to be always growing, even if quantitative data are not currently available on which to be able to further reflect.

## Discussion

If we talk about rhoticity and non-rhoticity, we immediately have in mind the studies of Labov (1972), on which a great deal has been written and said, so much so that on several occasions his own research techniques have been used; in this regard we recall the research of Mather (2012), or the study by Villarreal et al. (2020).

The aspect that we intend to investigate at this juncture, on the other hand, concerns the sociological reasons, properly so called, for which speaking subjects prefer to pronounce a letter or not as a distinctive, differential factor.

Social differentiation can be defined as the process by which the components of a population or a collectivity, be it a society, an association, an organization, a group, i.e., a social system, gradually acquire a distinct identity in terms of function, activity, structure, culture, authority, power, or other socially significant and relevant characteristics. In summary, social differentiation means becoming different in the light of social categories and for social causes. By extension, the outcome or progress of the social differentiation process by a given community is also called social differentiation (Gallino, 2014, p. 222).

Social differentiation thus becomes the tool at the service of society to guarantee distinctions and divisions. It is by its very nature that it uses pragmatic elements such as language to express these needs for distinction. Being rhotic or non-rhotic, thus, becomes clear proof of the Symmellian assumptions, according to which the human being is a differential being, since “as we never perceive the absolute magnitude of a stimulus, but only its difference with respect to the state of sensations as it has been given up to now, also our interest is not inherent in those vital contents which have always and everywhere been the diffused and general contents, but those by which each one is distinguished from the other. The common foundation on which all that is individual is built is something obvious, and therefore cannot require particular attention, which is, if anything, entirely consumed by individual differences. In fact, all practical interests, all the determinations of our position in the world, all the uses of other men are based on these differences between man and man, while the common ground in which all these processes take place is a constant factor that our conscience can neglect, because it affects all differences in the same way: and only these are important” (Bilotta, 2017, p.85).

As in a nutshell, societies feel the need, which starts with the individual, to affirm their difference based on the uniqueness that is sanctioned by symbolic and cultural tools such as the language.

Here, then, is the reason for the dual mechanism between the use or not of the r, because even a letter is a differential instrument, and therefore allows to perform “the cultural act par excellence—which—consists in drawing a line which produces a separate and delimited space, like the nemus, a sacred wood offered to the gods, the templum, a boundary enclosure for the gods, or simply the house with the threshold, limen” (Bourdieu, 1980, p. 318).

A letter becomes the border, the threshold with which to guarantee diversity, and above all, inequality.

It is for these reasons that the topic studied by Labov is fully part of the analysis of social classes, that is, one of the cornerstones of sociology.

First of all, by social class we mean a “complex of individuals”, who are in a similar position in the historically determined structure of the fundamental political and economic relations of a society, or who perform a similar function in the global organization of it. In particular, the proof of this is an important variable as the socio-economic status because it influenced in many ways the people also in the pronunciation. “The social status is probably the most crucial factor in the vocalization of /r/, as middle-class speakers are basically rhotic and most of the time produce an articulated /r/, while the rhotic is frequently vocalized by working-class speakers, especially the young ones” (Jauriberry, 2020, p. 6).

For these reasons the boundary between the classes is categorical, being univocally determined by the criterion assumed as the foundation or basis of the social class, so that each individual belongs to a single class and to only one; the sociologist underlines how “a social class is distinguished by its foundation (or base), that is the objective mechanism, which from the observer's point of view distributes individuals into different ensembles including very similar ways of a variable or a combination of variables.” (Gallino, 2014, p. 116); and again, it reminds us of what class sizes are, that is wealth, power and prestige.

Definitions, which become very useful for our discussion, as the question of rhoticity is clearly based on a series of boundaries which are variably “categorical”; this is because each class has very specific rules with which to sanction the differentiation; from the point of view of the internal aspects, concerning the individuals who constitute it, and the external aspects on society.

This is why social classes are considered as “communities of destiny” or to put it in the words of Weber (1922, p. 5) “possibility of life” why? Because a social class becomes the determining factor of the various types of behaviors and social actions such as language, because only when a certain subject belongs to it does it acquire a certain class consciousness, and therefore a certain vision of the surrounding world that necessarily requires, too, and above all, a specific method of communication.

Class, language and social differentiation are only the terms of an inseparable social equation, “There is in fact no difference in language, religion, customs, ideology, associative affiliation, work, skills, education, etc. which does not give rise to some type of differentiation in the form of doing, of being, of duty or of having within the population or sub-population that manifests them” (Gallino, 2014, p. 119).

In these terms, social differentiation linked to language belongs to the category of differences of being, therefore of the way of being and interacting with each other in a purely cultural context.

In all the dynamics of class (and therefore of power) that we have mentioned up to now, rhoticity and non-rhoticity seem to confirm the socio-anthropological conception according to which communication/language can be considered as an exchange of values. For example, Dickson and Hall-Lew (2017), have established the relation between class, gender, and rhoticity: this is the proof that exist an important bond between all these differentiation variables.

It is in this direction that Engels himself in “The condition of the working class in England”(1845), captures the aspects we have discussed up to now, even if, in food terms, which however have an intimate connection with the process of social differentiation like the question of rhoticity.

In the case of rhoticity/non-rhoticity, these are real class meanings, dictated by social trends, which allows members of the various classes to recover their rank and, if they can participate in the goods of society in equal measure and in the same way those who, like him, aspire to it, will realize that they have not lost it. At the same time, he will feel he must extend this conquest to the whole family, making sure that the impression of achieving or maintaining a certain social rank is shared by all (Halbwachs, 1913, p. 126).

Even more interesting, in this regard, is what Bourdieu argued, that is, in fact taste functions as a kind of sense of one's place, by which it orients those who occupy a certain place in the social space toward the social positions suited to their properties, toward the practices or toward the goods which suit those who occupy that position, which “are good” for them. The taste, we could say, even to hear or not a letter can, indeed almost certainly is so, arises as the sense of one's place for the different social groups that use the letter r or not; this is because, we reiterate it again, in emphasizing the differences between groups, cultures, social strata, and serves to strengthen group identity, to separate and distinguish “we” from “others” (ibidem).

The question becomes even more interesting, if we correlate these assumptions with what was first argued by Labov, and then by many scholars such as Trudgill and Hannah (2013, p. 13) or Dickson and Hall-Lew (2016), on social stratification and the pronunciation or not of the /r/: that is, what hovers, under the purely sociological profile is the affirmation of prestige, and therefore of that differential evaluation with which a higher or lower social position is attributed. Prestige, mediated from a linguistic point of view, is thus linked to the concept of wealth and power, which in turn constitute the three foundations of the social status, and therefore of the position that each subject occupies in society, and therefore the starting point for the social stratification process.

The semantic aspect underlined by Gallino (2014, p. 523) is interesting, according to which in the Anglo-Saxon anthropological and sociological literature the term status is used as a synonym of prestige, who knows that precisely this linguistic use is not at the basis of the entire process that has led to the dichotomy between rhotic and non-rhotic, in the sense that if prestige is a synonym of status, recourse to the pronunciation of r or not, would be a pragmatic declination of rational “social thought” with respect to the purpose, as Weber would say, which is hides behind this differential phenomenon; to use Cavalli's language, it would seem present, since the affirmation of the dichotomy between rhotic and non-rhotic, a form of naive sociology or a sort of innate sociological instinct with which each of us, regardless of their status, etc., structure observations, actions and elements to understand and observe the surrounding world.

If we associate with these phenomena, the trend documented by Dickson and Hall-Lew (2017), of the so-called derhoticization (it refers at the mechanism reduction of vocalization of /r/ of specific class (Jauriberry, 2020, p. 6), the considerable increase in non-rhoticity in the working class, already identified by Becker in 2009, brings to mind the imitative mechanisms of social classes and/or marginal ethnic groups, which are reminiscent in all respects of the “theory of the leisure class” proposed by Veblen (1934) according to which the less well-off social classes would tend to imitate the more well-to-do in order to try to assert their social position through prestigious instruments that in the original vision of Veblenian theory would be purely material, in our theoretical vision it is of a linguistic type. In this case, for example, the Veblenian theory has been demonstrated by the research work by Dickson and Hall-Lew (2016) in particular these researchers have discovered a strong correlation between the phenomenon in question, social class to which they belong and even gender.

Furthermore, this aspect is connected to the so-called social mobility, as already mentioned by Labov (1966), that is to the phenomenon of “displacement” from one social class to another, or at least, in the case of imitative mechanisms, an attempt to make this more or not as possible.

The question posed in these terms would seem to suggest a new perspective according to which the non-gearing of the wealthy classes would be a more immediate and tangible way than any other differential object, language being that which anticipates the same comfortable habitus: through the lack of pronunciation of /r/, the process of differentiation is catalyzed and consequently the borders, the regionalization of first social and then physical spaces, with which, alongside the differentiation, the inequality that more or less implicitly these processes intend to consolidate develops, to the point of engulfing.

In particular the relation between social class, mobility and the dichotomy “rhocity vs non-rhoticity” are related with “globalization, standard language multilingualism has become more respectable—positioning an expanded range of bilingual repertoires as (cosmopolitan) posh” (Rampton, 2018, p. 120).

## Concluding Remarks

From what we have been able to analyze up to now, the dichotomy “rhocity vs non-rhoticity” would require further study on the historical-social aspects. All this leads us to remember what Berger and Luckman argued, namely the fact that language constructs real buildings of symbolic representations; but it is not only capable of constructing such symbols but is more than anything else capable of creating semantic fields or zones of meaning (Beger and Luckman, 1989). With this article is clear that this dichotomy is very heterogeneous (Howson and Monahan, 2019, p. 26) and complex. But the question that revolves around rhoticity reminds us how language makes any type of social action present, socially relevant and highly variable. Only any qualitative approaches could reveal aspects that are still hidden. How much did class / status / power reasons affect pronunciation? Who could make further contributions? Only a multidisciplinary vision could clarify and shed new light on such fascinating issues.

## Author Contributions

DC and RS contributed to conception and design of the study, searched for relevant articles, wrote the first draft of the manuscript, wrote all the sections of the manuscript, and revised the manuscript critically. All authors contributed to manuscript revision, read, and approved the submitted version.

## Conflict of Interest

The authors declare that the research was conducted in the absence of any commercial or financial relationships that could be construed as a potential conflict of interest.

## Publisher's Note

All claims expressed in this article are solely those of the authors and do not necessarily represent those of their affiliated organizations, or those of the publisher, the editors and the reviewers. Any product that may be evaluated in this article, or claim that may be made by its manufacturer, is not guaranteed or endorsed by the publisher.
